# Molecular Identification of *Clinostomum chaacci* (Digenea: Clinostomidae) Metacercariae Reveals Two New Siluriform Hosts in the Peruvian Amazon

**DOI:** 10.3390/ani16213417

**Published:** 2026-10-29

**Authors:** Nicole Huamán, Nicolas Tarmeño, Ruperto Severino, David Lopez, Luis A. Ñacari, Jefferson Yunis-Aguinaga, Celso L. Cruces, Germán Augusto Murrieta-Morey, Jhon D. Chero

**Affiliations:** 1 Laboratorio de Zoología de Invertebrados, Departamento Académico de Zoología, Facultad de Ciencias Biológicas (FCB), Universidad Nacional Mayor de San Marcos (UNMSM), Av. Universitaria Cruce con Av. Venezuela Cuadra 34, Lima 15081, Peru; nicole.huaman@unmsm.edu.pe (N.H.); ntarmenor@unmsm.edu.pe (N.T.); rseverinol@unmsm.edu.pe (R.S.); david.lopez1@unmsm.edu.pe (D.L.); jcherod@unmsm.edu.pe (J.D.C.); 2 Laboratorio de Ecología y Evolución de Parásitos, Facultad de Ciencias del Mar y Recursos Biológicos, Universidad de Antofagasta, Antofagasta 1270300, Chile; luis.nacari.enciso@gmail.com; 3 Facultad de Ciencias Veterinarias y Biológicas, Universidad Científica del Sur, Carretera Panamericana Sur, Km 19, Lima 15001, Peru; 4 Laboratorio de Biología Celular y Molecular, Facultad de Medicina Humana, Universidad Ricardo Palma (URP), Av. Alfredo Benavides 5440 Santiago de Surco, Lima 15039, Peru; celso.cruces@urp.edu.pe; 5 Laboratorio de Parasitología y Sanidad Acuícola, Instituto de Investigaciones de la Amazonia Peruana (IIAP), Carretera Iquitos-Nauta, Km 4.5, Iquitos 16001, Loreto, Peru; gmurrieta@iiap.gob.pe; 6 Departamento Académico de Hidrobiología, Facultad de Ciencias Biológicas (FCB), Universidad Nacional de la Amazonía Peruana (UNAP), Carretera Zúngaro Cocha, Iquitos 16001, Loreto, Peru

**Keywords:** Amazon basin, Clinostomidae, *Pimelodella cristata*, *Rineloricaria morrowi*, molecular identification, 28S rDNA, *Cox1*

## Abstract

*Clinostomum chaacci* Sereno-Uribe, López-Jiménez, González-García, Ortega-Olivares & García-Varela, 2025 is a recently described clinostomid trematode whose metacercariae have been molecularly identified in freshwater fishes from Mexico and South America. In October 2025, 29 siluriform fishes, comprising 18 *Pimelodella cristata* (Müller & Troschel, 1849) (Heptapteridae) and 11 *Rineloricaria morrowi* Fowler, 1940 (Loricariidae), were examined from the Nanay River, Loreto, Peru. Eight metacercariae were found encysted in the cephalic integument. The specimens were characterized morphologically and identified through Bayesian phylogenetic analyses of the mitochondrial *Cox1* gene and the nuclear 28S rDNA. The new *Cox1* sequences from both hosts were identical and nested within the *C. chaacci* clade, differing by 0.42–5.27% from conspecific isolates, whereas the 28S rDNA sequence differed by only 0–0.08% from reference sequences of *C. chaacci*. Morphometric data partially overlapped with those of *C. chaacci* metacercariae previously reported from Peru. The records from *P. cristata* and *R. morrowi* represent two new second intermediate hosts and add Loricariidae to the known host spectrum of *C. chaacci*, providing further evidence that a taxonomically diverse assemblage of fishes participates in its transmission in the western Amazon Basin.

## Simple Summary

Trematodes of the genus *Clinostomum* use freshwater snails as first intermediate hosts, fishes or amphibians as second intermediate hosts, in which their larval stages (metacercariae) form cysts, and fish-eating birds as definitive hosts. Because the metacercariae of closely related species look very similar, DNA sequences are needed to identify them reliably. In this study, we examined two catfish species from the Nanay River in the Peruvian Amazon, *Pimelodella cristata* and *Rineloricaria morrowi*, and found *Clinostomum* metacercariae encysted on the head. Using two genetic markers, we identified them as *Clinostomum chaacci*, a species formally described in 2025. These findings add two new hosts for this parasite, including the first record in a loricariid catfish, and show that a wide variety of fishes participate in its transmission in the Amazon.

## 1. Introduction

Species of *Clinostomum* Leidy, 1856 (Digenea: Clinostomidae) are trematodes with complex life cycles. Cercariae released from freshwater snails, the first intermediate hosts, penetrate fishes or amphibians, in which they develop into encysted metacercariae, commonly known as “yellow grubs”. The life cycle is completed when piscivorous birds, mainly ardeids, ingest infected second intermediate hosts, and the adults establish in the oral cavity or oesophagus [1,2,3]. Encysted metacercariae may cause pathological alterations in the tissues of their fish hosts [4]. However, reliable species-level identification of these larval stages is difficult, because metacercariae of closely related species share a similar morphology and lack fully developed reproductive structures. Consequently, DNA sequence data, particularly from the mitochondrial *Cox1* gene and nuclear rDNA, have become essential for identifying *Clinostomum* metacercariae and linking them with their adult stages [2,3].

*Clinostomum chaacci* Sereno-Uribe, López-Jiménez, González-García, Ortega-Olivares & García-Varela, 2025 was recently described from adult specimens collected from the oral cavity of two ardeid birds in southeastern Mexico, namely the great blue heron, *Ardea herodias* Linnaeus, 1758 (type host), and the bare-throated tiger-heron, *Tigrisoma mexicanum* Swainson, 1834 [1]. Before its formal description, this species had been recognized as *Clinostomum* sp. lineage 3 in molecular surveys conducted in Mexico and Middle America [1,2]. The examination of adult specimens, together with molecular data, allowed this previously unnamed lineage to be formally described as *C. chaacci*. Phylogeographic analyses based on *Cox1* further revealed slight genetic differentiation among populations of this species, suggesting reduced connectivity and a subdivision into geographically structured populations [1].

In Mexico, metacercariae of this species have been recorded from the heptapterid catfishes *Rhamdia guatemalensis* (Günther, 1864) and *Rhamdia rogersi* Regan, 1907, and from the gobiiform fish *Sicydium salvini* Ogilvie-Grant, 1884 and *Gobiomorus maculatus* (Günther, 1859) [2,3]. In South America, genetically conspecific metacercariae have been reported from the cichlid fishes *Crenicichla vittata* Heckel, 1840 and *Cichlasoma dimerus* (Heckel, 1840), and from the heptapterid catfish *Pimelodella laticeps* Eigenmann, 1917 in Argentina [4,5]. More recently, analyses of *Cox1* and 28S rDNA sequences confirmed the occurrence of *C. chaacci* in the callichthyid catfish *Hoplosternum littorale* (Hancock, 1828) from Iquitos, Loreto, in the Peruvian Amazon, representing the first molecularly confirmed record of this species in Peru and contributing to filling the geographic gap between its previously documented occurrences in Mexico and Argentina [6].

During a parasitological survey of freshwater fishes from the Peruvian Amazon, *Clinostomum* metacercariae were recovered from two siluriform species not previously reported as hosts of these parasites. Therefore, the objectives of the present study were: (i) to characterize these metacercariae morphologically and identify them using molecular data from the mitochondrial *Cox1* gene and nuclear 28S rDNA; (ii) to assess their phylogenetic relationships with previously characterized isolates of *Clinostomum*; and (iii) to expand current knowledge of the host range and geographical distribution of *C. chaacci*.

## 2. Materials and Methods

### 2.1. Sample Collection and Morphological Study

**Figure 1:**
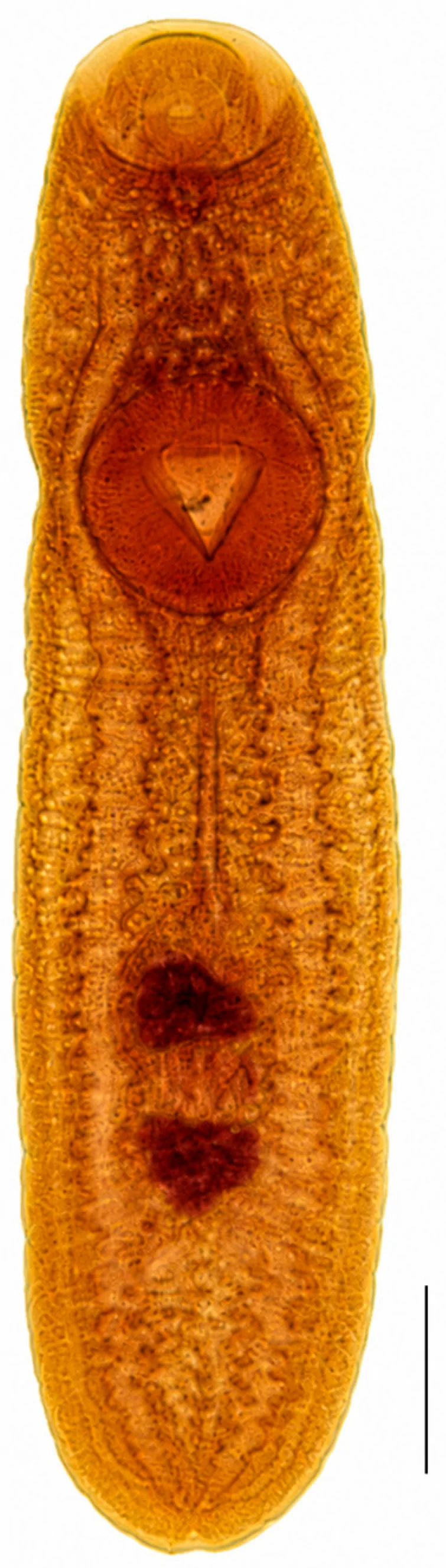
Metacercaria of *Clinostomum chaacci* Sereno-Uribe, López-Jiménez, González-García, Ortega-Olivares & García-Varela, 2025 isolated from *Pimelodella cristata* (ventral view). Scale bar = 500 µm.

In October 2025, a total of 29 siluriform fishes, comprising 11 specimens of *Rineloricaria morrowi* Fowler, 1940 and 18 specimens of *Pimelodella cristata* (Müller & Troschel, 1849), were collected by local fishermen using gillnets in the Nanay River (3°47′ S, 73°22′ W), near Santa Clara Beach, Loreto Region, Peru. Fish specimens were identified by researchers from the Instituto de Investigaciones de la Amazonía Peruana (IIAP), Loreto, Peru, and voucher specimens were deposited in the Ichthyological Collection of IIAP (CIIAP) under the accession numbers CIIAP 6774 for *P. cristata* and CIIAP 6775 for *R. morrowi*. A total of eight metacercariae were recovered from the examined fish. Of these, five metacercariae from *P. cristata* were used for morphological examination (Figure 1), while one metacercaria from each host species was selected for DNA extraction and sequencing (isolate 630 from *P. cristata* and isolate 631 from *R. morrowi*). The second metacercaria from *R. morrowi* was also processed for DNA extraction, but no sequences were obtained because the sample was contaminated. The collection, fixation, preservation, clearing, and morphological preparation of metacercariae followed the protocols described by Chero et al. [6] and Huaman et al. [7]. Morphological examinations and photomicrography were performed using a Nikon™ Eclipse Ni-L compound microscope (Nikon Corporation, Tokyo, Japan) equipped with bright-field and differential interference contrast (DIC) optics. All measurements were obtained from five specimens (*n* = 5) and are presented in micrometers, unless otherwise indicated, as ranges followed by means in parentheses. Measurements represent straight-line distances between the extreme points of the structures examined. Hologenophores and voucher specimens were deposited in the Helminthological Collection of the Museum of Natural History at San Marcos University (MUSM-HEL), Lima, Peru.

### 2.2. Molecular Analysis

Genomic DNA of the metacercariae was extracted using a TIANamp Genomic DNA Kit (Tiangen Biotech, Beijing, China) following the manufacturer’s protocol. The partial 28S rDNA was amplified by polymerase chain reaction (PCR) using the forward primer digl2 (5′-AAG CAT ATC ACT AAG CGG-3′) and the reverse primer 1500R1 (5′-GCT ACT AGA TGG TTC GAT TAG-3′) [8]. The partial mitochondrial cytochrome c oxidase subunit I gene (*Cox1*) was amplified using the forward primer Cox 5_Schisto (5′-TCT TTR GAT CAT AAG CG-3′) and the reverse primer JB5 (5′-AGC ACC TAA ACT TAA AAC ATA ATG AAA ATG-3′) [9,10,11]. PCR assays were carried out in a total volume of 25 µL containing 12.5 µL of PCRBIO Taq Mix Red (PCR Biosystems Ltd., London, UK), 0.3 µL of each primer (10 µM), 1.2 µL of DNA template and ultrapure water to the final volume.

Thermocycling conditions consisted of an initial denaturation at 95 °C for 1 min, followed by 35 cycles of denaturation at 95 °C for 15 s, annealing at 50 °C (28S rDNA) or 48 °C (*Cox1*) for 20 s, and extension at 72 °C for 30 s, with a final extension at 72 °C for 1 min. PCR products were purified with sparQ PureMag Beads (Quantabio, Beverly, MA, USA) following the manufacturer’s instructions and sequenced by PSOMAGEN Inc. (Brooklyn, NY, USA) using the Sanger method.

Sequences were edited and assembled into consensus contigs using Geneious Prime v2026.0.2 (Biomatters Ltd., Auckland, New Zealand). The newly generated sequences were deposited in GenBank under the accession numbers QB134948 and QB134949 (*Cox1*) and QB131873 (28S rDNA). Although both isolates were submitted for 28S rDNA sequencing, the sample of isolate 631 (*R. morrowi*) was contaminated; therefore, only the 28S rDNA sequence of isolate 630 (*P. cristata*) was obtained and deposited. Homologous sequences were retrieved from the National Center for Biotechnology Information (NCBI) GenBank database using the Basic Local Alignment Search Tool (BLAST+). The newly generated 28S rDNA and *Cox1* sequences were aligned together with representative sequences of Clinostomidae using the MUSCLE algorithm implemented in MEGA v12 [12]. For the *Cox1* alignment, sequences of *Euclinostomum* spp. and *Ithyoclinostomum* spp. (Clinostomidae) and of *Alaria mustelae* Bosma, 1931 (Diplostomidae) were selected as outgroup taxa, following previous phylogenetic studies of *Clinostomum* [6]. Similarly, outgroups for the 28S rDNA dataset included representatives of *Euclinostomum* spp., *Ithyoclinostomum* spp., *Odhneriotrema incommodum* (Leidy, 1856) (Clinostomidae) and *A. mustelae*. The sequences of *C. chaacci* used for comparison are listed in Table 1, and the complete list of sequences included in the phylogenetic analyses, with their hosts, localities and references, is provided in Table S3 [13,14,15,16,17,18,19,20,21,22,23,24,25,26,27,28,29,30,31,32,33,34,35,36,37,38,39,40,41,42].

### 2.3. Phylogenetic Analyses and Genetic Distances

Bayesian phylogenetic analyses were performed in MrBayes v3.2 [43] implemented in Geneious. For the 28S dataset, the best-fitting nucleotide substitution model (GTR+G) was selected under the Bayesian Information Criterion (BIC) using jModelTest [44]. Bayesian inference employed six substitution types (nst = 6) with gamma-distributed among-site rate variation (rates = gamma; four discrete gamma categories) and unequal base frequencies estimated under a Dirichlet prior (statefreqpr = dirichlet(1, 1, 1, 1)). Two independent Markov chain Monte Carlo (MCMC) runs, each consisting of four chains, were conducted for 1,000,000 generations, sampling every 1000 generations. Convergence between runs was assessed using the average standard deviation of split frequencies (ASDSF < 0.02) together with effective sample size (ESS) values exceeding 200 for all parameters. The first 25% of sampled trees were discarded as burn-in, and the remaining trees were used to construct a 50% majority-rule consensus tree with Bayesian posterior probabilities (PP) mapped onto the nodes.

Phylogenetic inference of the mitochondrial *Cox1* dataset was conducted under a partitioned scheme based on codon positions. Prior to the analyses, the alignment was inspected to confirm the absence of stop codons and frame shifts under the invertebrate mitochondrial genetic code, thereby ensuring that all sequences represented functional protein-coding regions and that codon positions were correctly assigned. The alignment was partitioned into first, second, and third codon positions. The optimal partitioning scheme and substitution models were selected using ModelFinder [45] as implemented in IQ-TREE [46], which identified TN+F+I+G4, K3Pu+F+G4, and TIM3+F+R3 as the best-fitting models for the first, second, and third codon positions, respectively. Because these models are not implemented in MrBayes, each partition was analysed under the general time-reversible model (GTR), with independent estimation of substitution parameters, base frequencies, gamma shape, and proportion of invariant sites for each codon position (unlink statefreq, revmat, shape, pinvar), while branch lengths were linked across partitions and relative substitution rates among partitions were allowed to vary (ratepr = variable). Two independent MCMC analyses with four chains each were run for 2,000,000 generations, sampling every 1000 generations. Convergence was assessed using the average standard deviation of split frequencies (ASDSF < 0.02) and ESS values greater than 200. The first 25% of sampled trees were discarded as burn-in, and the remaining trees were summarized as a 50% majority-rule consensus tree with posterior probabilities.

Pairwise genetic divergences were calculated as uncorrected p-distances, expressed as percentages, using MEGA v12 [12], with gaps and missing data treated by pairwise deletion. The distance matrices (Tables S1 and S2) were computed from alignments of 471 (*Cox1*) and 1223 (28S rDNA) nucleotide positions.

## 3. Results

Overall, eight encysted *Clinostomum* metacercariae were recovered from the cephalic integument, mainly on the dorsal and lateral surfaces of the head: six from *P. cristata* and two from *R. morrowi*. In *P. cristata*, six metacercariae were recovered from five of the 18 examined fish, corresponding to a prevalence of 27.8% and a mean intensity of 1.2 metacercariae per infected fish. In *R. morrowi*, two metacercariae were recovered from one of the 11 examined fish, corresponding to a prevalence of 9.1% and a mean intensity of 2.0 metacercariae per infected fish.

### 3.1. Morphological Characterization of the Metacercariae

This was based on five metacercariae from *P. cristata*; *n* = 5 for all measurements; Figure 1. Body robust, linguiform, 3.95–4.76 (4.44) mm long, with two constrictions at ventral sucker level; greatest width 0.97–1.08 (1.03) mm at testicular level. Body length/body width ratio 3.9–4.7 (4.3). Forebody 1.54–1.74 (1.66) mm long. Hindbody 2.41–3.01 (2.78) mm long. Oral sucker 222–273 × 209–258 (253 × 241). Oral sucker width/body width ratio 0.21–0.26 (0.23). Pharynx 120–150 × 137–158 (139 × 149). Intestinal caeca diverticulate. Ventral sucker 624–685 × 627–689 (658 × 659), with triradiate aperture. Ventral sucker width/body width ratio 0.59–0.67 (0.64). Ventral sucker width/oral sucker width ratio 2.49–3.08 (2.74). Testes two, tandem, asymmetrical, lobulated; anterior testis, 216–332 × 273–419 (289 × 324), anterior testis width/anterior testis length ratio 1.08–1.26 (1.19); posterior testis 241–297 × 319–440 (261 × 369), posterior testis width/posterior testis length ratio 1.30–1.75 (1.47). Cirrus sac, kidney-shaped, in dextrolateral to anterior testis, 259–294 × 166–213 (276 × 183), cirrus sac length/body length ratio 0.05–0.07 (0.06). Ovary small, intertesticular and dextral, 114–170 × 72–89 (141 × 81), ovary width/ovary length ratio 0.52–0.63 (0.58). Uterine sac tubular, between genital complex and ventral sucker.

Hosts of the present study: *Pimelodella cristata* (Müller & Troschel, 1849) (Heptapteridae) and *Rineloricaria morrowi* Fowler, 1940 (Loricariidae).

Site of infection: Cephalic integument.

Locality: Nanay River (3°47′ S, 73°22′ W), near Santa Clara Beach, Loreto Region, Peru.

Specimens deposited: 3 voucher specimens (MUSM XXXX).

Representative DNA sequences: QB131873 (28S rDNA from *P. cristata*, 1212 bp), QB134948 (*Cox1* mDNA from *P. cristata*, 1159 bp) and QB134949 (*Cox1* mDNA from *R. morrowi*, 1147 bp).

### 3.2. Remarks

The metacercariae identified as *C. chaacci* from *P. cristata* in the present study showed partial morphometric agreement with those previously reported as *C. chaacci* from *H. littorale* by Chero et al. [6] (see Table 2). Body length overlapped considerably (3950–4760 vs. 4100–6500 µm), although the present specimens were narrower (970–1080 vs. 1200–2000 µm) and proportionally more elongate (BL/BW = 3.9–4.7 vs. 3.14–3.25). They also exhibited a larger pharynx (120–150 vs. 98–120 µm) and a shorter cirrus sac (259–294 vs. 390–400 µm). Nevertheless, several characters, including sucker dimensions, testicular measurements, ovarian dimensions, and proportional indices, showed overlapping ranges. These morphometric differences are interpreted as intraspecific variation, consistent with the molecular identification of the present specimens as *C. chaacci*.

### 3.3. Molecular Analyses

**Figure 2:**
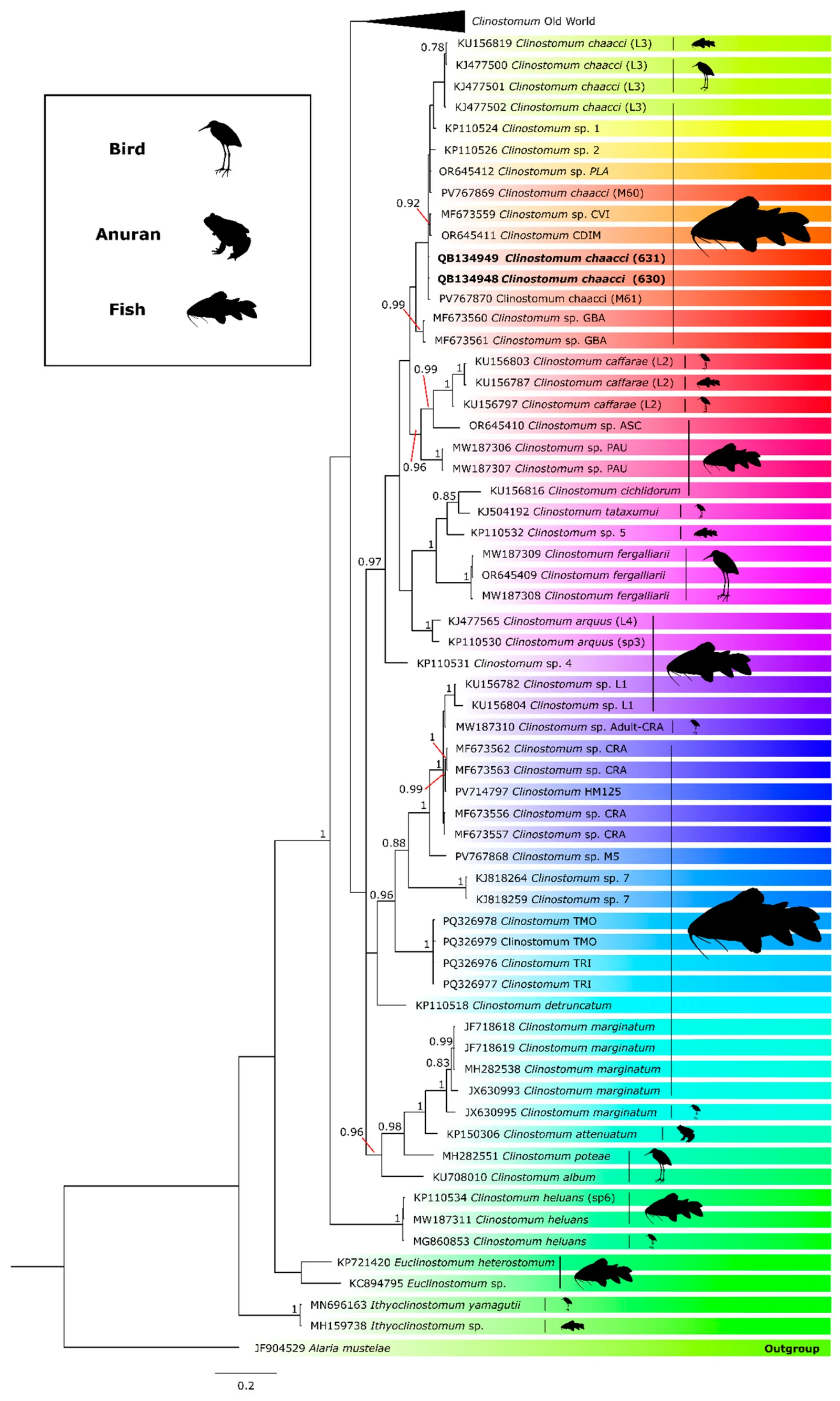
Phylogenetic tree of species/lineages of clinostomids based on the *Cox1* gene inferred using Bayesian inference (BI). Numbers at nodes are Bayesian posterior probabilities. Sequences of *Clinostomum chaacci* obtained in this study are in black boldface. Vertebrate silhouettes indicate the host group from which the corresponding parasite material was isolated. Coloured backgrounds indicate different lineages. Scale-bar: average number of nucleotide substitutions per site.

The phylogenetic analysis based on the *Cox1* dataset recovered the two newly generated sequences within a clade comprising all isolates assigned to *C. chaacci* (Table 1), with a Bayesian posterior probability of 0.76 (Figure 2). The newly generated sequences from *P. cristata* (QB134948) and *R. morrowi* (QB134949) were identical to each other and differed by 0.42–5.27% from the previously characterized isolates of *C. chaacci*. The lowest divergences were observed with the Peruvian isolates from *H. littorale* (PV767870 and PV767869; 0.42–1.06%) and with the Argentine isolates MF673559, OR645412 and OR645411 (0.84–0.90%), whereas divergence from the Mexican isolates ranged from 2.11 to 5.27%. The overall intraspecific divergence within *C. chaacci* ranged from 0 to 5.70%. The *C. chaacci* clade was recovered as sister to *Clinostomum* sp. GBA (MF673560 and MF673561), with interlineage divergence of 4.85–8.65%, and the lowest divergence from a nominal congener was observed with *C. arquus* (7.59–11.39%). Pairwise distances are provided in Table S1.

**Figure 3:**
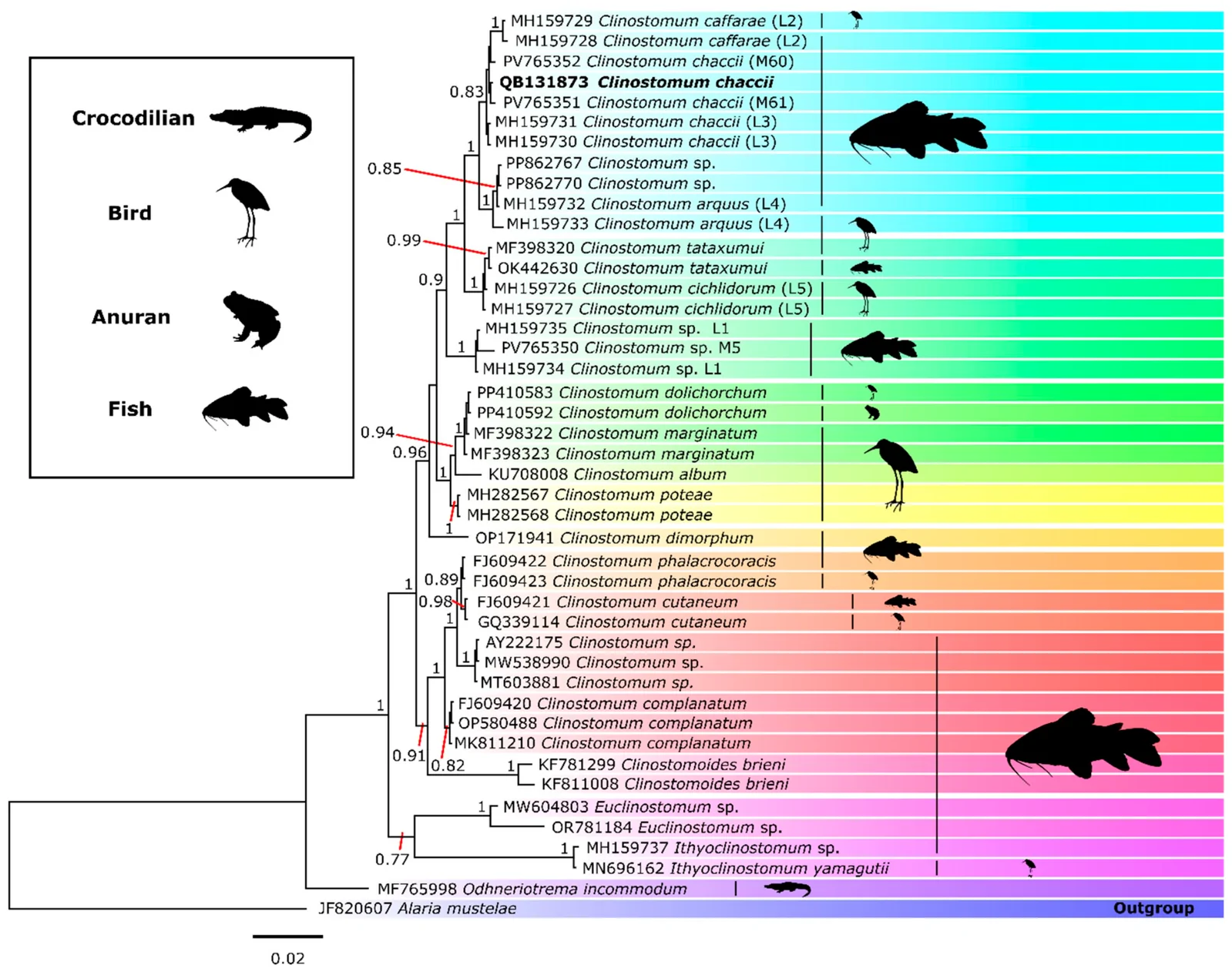
Phylogenetic tree of species/lineages of clinostomids based on 28S rDNA inferred using Bayesian inference (BI). Numbers at nodes are Bayesian posterior probabilities. The sequence of *Clinostomum chaacci* obtained in this study is in black boldface. Vertebrate silhouettes indicate the host group from which the corresponding parasite material was isolated. Coloured backgrounds indicate different lineages. Scale-bar: average number of nucleotide substitutions per site.

The phylogenetic analysis based on the 28S rDNA dataset recovered the newly generated sequence (QB131873) together with the available sequences of *C. chaacci* (PV765351, PV765352, MH159730, and MH159731) in a clade with a Bayesian posterior probability of 0.83 (Figure 3), closely related to *C. caffarae*. The new sequence was identical to MH159730 and MH159731, obtained from metacercariae of *R. guatemalensis* and *G. maculatus* in Mexico, and differed by 0.08% from PV765351 and PV765352, obtained from *H. littorale* in Peru. Intraspecific divergence among all *C. chaacci* sequences ranged from 0 to 0.17%, whereas divergence from the other nominal species of *Clinostomum* ranged from 0.33 to 4.62%, with the lowest values observed with *C. caffarae* (0.33–0.41%). Pairwise distances are provided in Table S2.

## 4. Discussion

The molecular evidence obtained in the present study confirmed that the *Clinostomum* metacercariae recovered from *P. cristata* and *R. morrowi* correspond to *C. chaacci*. The newly generated sequences from both hosts were consistently associated with previously characterized isolates [1,6] of this species in the phylogenetic analyses based on both 28S rDNA and mitochondrial *Cox1*, and the morphometric features of the metacercariae from *P. cristata* partially overlapped with those of conspecific metacercariae from *H. littorale* (Table 2). Species-level identification of *Clinostomum* metacercariae based exclusively on morphology is often difficult because reproductive structures are not fully developed and the morphological characters available at the larval stage may be insufficient to reliably distinguish closely related species [2,47,48]. This issue is particularly relevant in the Peruvian Amazon, where some metacercarial records remain based exclusively on morphological identification [49]. For instance, *C. marginatum* (Rudolphi, 1819) metacercariae were recently reported from *Poecilia reticulata* Peters, 1859 in Iquitos based on morphological characters but without molecular corroboration [50]. Consequently, molecular data have become essential for distinguishing closely related species and genetically distinct lineages [2,16,51]. In the present study, the two molecular markers showed different levels of genetic variability but provided complementary evidence for species identification: 28S rDNA was highly conserved among conspecific isolates, whereas *Cox1* exhibited considerably greater variation. This difference is consistent with previous studies showing greater discriminatory power of *Cox1* relative to the more conserved 28S rDNA marker in digeneans [6,32]. Although the posterior probabilities supporting the *C. chaacci* clade were moderate in both analyses, the new sequences showed minimal divergence from conspecific isolates, particularly those from Peru and Argentina. Thus, the taxonomic assignment of the present metacercariae is supported by the congruent phylogenetic and genetic evidence provided by both nuclear and mitochondrial markers. Nevertheless, these conclusions are based on a limited number of newly generated sequences (two *Cox1* sequences and one 28S rDNA sequence), which does not allow the genetic variability of *C. chaacci* in the study area, or potential differences between parasites from different host species, to be assessed. Additional molecular data from a larger number of specimens and hosts will therefore be necessary to address these aspects.

The *Cox1* dataset also revealed considerable mitochondrial variation within *C. chaacci*, with intraspecific divergence reaching 5.70%. Several of these isolates were originally reported under informal lineage designations, particularly *Clinostomum* sp. lineage 3 and related molecular lineages, before their association with the formally described *C. chaacci* was established [1,2,6]. Despite this relatively broad range of intraspecific divergence, the newly generated sequences remained nested within the *C. chaacci* assemblage. Moreover, the new sequences were genetically closest to other South American isolates from Peru and Argentina and more divergent from Mexican isolates. This pattern suggests a closer mitochondrial affinity among the South American isolates, although the geographically uneven sampling currently available for *C. chaacci* prevents firm conclusions regarding population structure. Broader geographical sampling will therefore be necessary to determine whether this pattern reflects genuine phylogeographic differentiation or incomplete sampling of the mitochondrial diversity of the species. Nevertheless, geographical structuring is plausible because Sereno-Uribe et al. [1] documented high haplotype diversity and partial genetic differentiation among populations of *C. chaacci* from Mexico and Middle America.

The relationship between *C. chaacci* and the unidentified lineage *Clinostomum* sp. GBA deserves separate consideration. GBA, originally characterized from metacercariae infecting *Gymnogeophagus balzanii* (Perugia, 1891) (Cichlidae) in Argentina [4], was recovered outside but closely related to the *C. chaacci* assemblage in the present *Cox1* phylogeny. The lower values of divergence between both groups overlapped with the upper limit of intraspecific variation detected within *C. chaacci*, whereas divergence from the closest nominal congener, *C. arquus* Sereno-Uribe, García-Varela, Pinacho-Pinacho & Pérez-Ponce de León, 2018, was higher. This overlap indicates that *Cox1* genetic distances alone may be insufficient for establishing species boundaries in *Clinostomum* and reinforces the need to evaluate mitochondrial divergence together with phylogenetic relationships, nuclear markers, and morphological evidence [2]. This is particularly relevant in *Clinostomum*, in which molecular surveys continue to reveal genetically distinct lineages that remain formally undescribed [4,5].

The occurrence of *C. chaacci* in *P. cristata* and *R. morrowi* represents two new second intermediate host records and further broadens the taxonomic spectrum of fishes involved in the life cycle of this trematode. Molecularly characterized metacercariae currently associated with *C. chaacci* have been reported from fishes belonging to several taxonomically distinct groups. Among Siluriformes, previous records include the heptapterids *R. guatemalensis*, *R. rogersi*, and *P. laticeps*, as well as the callichthyid *H. littorale* [2,5,6]. Records from other orders include the cichlids *C. vittata* and *C. dimerus* (Cichliformes: Cichlidae) and the gobiiforms *S. salvini* (Gobiidae) and *G. maculatus* (Eleotridae) [3,4,5,30]. The present study adds *P. cristata* (Siluriformes: Heptapteridae) and, importantly, *R. morrowi* (Siluriformes: Loricariidae), thereby adding Loricariidae to the known host spectrum of the species. Collectively, these records indicate that the known second intermediate host spectrum of *C. chaacci* spans multiple fish families and orders. Although this pattern may suggest low host specificity at the metacercarial stage, current host records are still limited and geographically uneven, and systematic sampling of local fish assemblages will be required to assess host specificity rigorously.

Metacercariae of *Clinostomum* have been reported from different microhabitats in fishes, including the integument, fins, musculature, and body cavity, indicating that sites of encystment may vary among host–parasite associations [52]. In the present study, metacercariae of *C. chaacci* recovered from *P. cristata* and *R. morrowi* were found encysted in the cephalic integument, mainly on the dorsal and lateral surfaces of the head. Previous records suggest some variation in the site of infection among second intermediate hosts. In the Peruvian Amazon, metacercariae of *C. chaacci* from *H. littorale* were found encysted in the musculature [6]. Similarly, the Argentine isolates *Clinostomum* sp. CVI from *C. vittata* and *Clinostomum* sp. PLA from *P. laticeps*, both currently associated with *C. chaacci*, were recovered from the mesentery and muscle fascia and from the musculature, respectively. In contrast, *Clinostomum* sp. CDIM from *C. dimerus* was recovered from the fins [4,5]. These observations suggest that *C. chaacci* may use different sites of encystment depending on the second intermediate host. However, because the site of infection has not been consistently documented for all molecularly characterized isolates, particularly those from Mexico and Middle America, additional comparative studies are required to determine whether this variation is associated with host identity or other ecological factors. Although histopathological analyses were not performed in the present study, Montes et al. [4] documented pathological alterations associated with *Clinostomum* metacercariae in Argentine freshwater fishes, including isolates currently assigned to *C. chaacci*, suggesting that the site of encystment may influence the type and extent of tissue damage in the host.

With respect to its geographical distribution, *C. chaacci* was recently confirmed from *H. littorale* in Loreto, representing the first molecular record of this species in the Peruvian Amazon [6]. Therefore, the present findings do not represent a major geographical range extension, but provide additional evidence that *C. chaacci* circulates among different second intermediate hosts in this region. Interestingly, genetically closely related isolates have now been recovered from *H. littorale*, *P. cristata*, and *R. morrowi* in Loreto, suggesting that several fish species may participate in the local transmission cycle of this trematode. These records, together with those reported from other fish hosts in Central and South America, indicate that the diversity of second intermediate hosts used by *C. chaacci* is probably greater than currently recognized. To our knowledge, the life cycles of clinostomids remain unstudied in the Peruvian Amazon, and local dietary data confirming the consumption of these fish hosts by definitive avian hosts are lacking. However, several piscivorous birds, including *Ardea cocoi* Linnaeus, 1766, *Ardea alba* Linnaeus, 1758, *Egretta thula* (Molina, 1782), *Butorides striata* (Linnaeus, 1758), *Pilherodius pileatus* (Boddaert, 1783), *Megaceryle torquata* (Linnaeus, 1766), and *Chloroceryle amazona* (Latham, 1790), have been recorded in the Nanay River basin [53]. Although direct predation on *P. cristata* and *R. morrowi* has not been documented locally, Dias et al. [54], in the upper Paraná River floodplain, Brazil, reported several siluriform fishes in the diet of piscivorous waterbirds. These included *Pimelodella gracilis* (Valenciennes, 1835), *Loricariichthys platymetopon* Isbrücker & Nijssen, 1979, and the armored catfishes *H. littorale* and *Trachydoras paraguayensis. Hoplosternum littorale*, which has also been reported as a second intermediate host of *C. chaacci* in Loreto [6], constituted an important prey item for piscivorous birds.

Some species of *Clinostomum*, particularly *C. complanatum* (Rudolphi, 1814), have been reported infecting humans following the consumption of raw or undercooked fish [55,56]. No human infection has been attributed to *C. chaacci*, and its zoonotic potential remains unknown. Among its known hosts in the Peruvian Amazon, *H. littorale* is consumed locally, whereas *R. morrowi* is mainly of ornamental interest; therefore, molecular surveys of metacercariae in fishes used for human consumption would help to clarify the potential public health relevance of this species.

Finally, the helminth fauna of wild birds in Peru remains poorly known, despite the important role that these vertebrates play as definitive hosts of numerous digenean trematodes [57,58]. Because adults of *Clinostomum* are commonly found in piscivorous birds, especially members of the Ardeidae [1], the examination of fish-eating birds in the Peruvian Amazon represents an important next step for linking metacercariae with their adult stages and clarifying the life cycle and transmission dynamics of *C. chaacci* in this region.

## 5. Conclusions

The integration of morphological data with mitochondrial (*Cox1*) and nuclear (28S rDNA) sequences allowed the identification of the *Clinostomum* metacercariae encysted in the cephalic integument of *Pimelodella cristata* and *Rineloricaria morrowi* from the Nanay River, Peruvian Amazon, as *Clinostomum chaacci*. These findings represent two new second intermediate host records for this trematode and the first record in a member of the family Loricariidae. They also add to the evidence that the metacercarial stage of *C. chaacci* can use a taxonomically diverse range of fishes, including several siluriform families. The close genetic similarity of the new isolates to other South American isolates, together with the considerable intraspecific *Cox1* divergence observed within the species, highlights the value of combining multiple molecular markers with phylogenetic analyses to identify *Clinostomum* larval stages, which cannot be reliably distinguished by morphology alone.

The occurrence of genetically closely related isolates of *C. chaacci* in three fish species from Loreto indicates that several hosts are involved in the local transmission of this parasite, and that its host range in the Amazon Basin is probably wider than currently recognized. Future studies should include broader and more systematic sampling of local fish assemblages to assess host specificity and population structure, histopathological evaluation of infected hosts, and the examination of piscivorous birds in the Peruvian Amazon to link metacercariae with adult stages and clarify the complete life cycle of *C. chaacci*. In addition, molecular surveys of fish species used for human consumption are recommended to evaluate the potential public health relevance of this trematode.

## Figures and Tables

**Table 1:** Sequences of *Clinostomum chaacci* (including isolates originally reported under informal lineage names) used in the present study. Newly generated sequences are in bold. The complete list of sequences used in the phylogenetic analyses is provided in Table S3.

Parasite Species/Isolate	Marker	Stage	Host Species	Locality	GenBank	Reference
***Clinostomum chaacci*** **(630)**	***Cox1***	**M**	***Pimelodella cristata***	**Peru**	**QB134948**	**Present study**
***Clinostomum chaacci*** **(631)**	***Cox1***	**M**	***Rineloricaria morrowi***	**Peru**	**QB134949**	**Present study**
*Clinostomum chaacci* (M60)	*Cox1*	M	*Hoplosternum littorale*	Peru	PV767869	[6]
*Clinostomum chaacci* (M61)	*Cox1*	M	*Hoplosternum littorale*	Peru	PV767870	[6]
*Clinostomum chaacci* (L3)	*Cox1*	M	*Rhamdia rogersi*	Mexico	KU156819	[2]
*Clinostomum chaacci* (L3)	*Cox1*	A	*Tigrisoma mexicanum*	Mexico	KJ477500	[2]
*Clinostomum chaacci* (L3)	*Cox1*	A	*Ardea herodias*	Mexico	KJ477501	[2]
*Clinostomum chaacci* (L3)	*Cox1*	M	*Rhamdia guatemalensis*	Mexico	KJ477502	[2]
*Clinostomum* sp. 1 ^a^	*Cox1*	M	*Rhamdia guatemalensis*	Mexico	KP110524	[3]
*Clinostomum* sp. 2 ^a^	*Cox1*	M	*Sicydium salvini*	Mexico	KP110526	[3]
*Clinostomum* sp. CVI ^a^	*Cox1*	M	*Crenicichla vittata*	Argentina	MF673559	[4]
*Clinostomum* sp. CDIM ^a^	*Cox1*	M	*Cichlasoma dimerus*	Argentina	OR645411	[5]
*Clinostomum* sp. PLA ^a^	*Cox1*	M	*Pimelodella laticeps*	Argentina	OR645412	[5]
***Clinostomum chaacci*** **(630)**	**28S rDNA**	**M**	***Pimelodella cristata***	**Peru**	**QB131873**	**Present study**
*Clinostomum chaacci* (M60)	28S rDNA	M	*Hoplosternum littorale*	Peru	PV765352	[6]
*Clinostomum chaacci* (M61)	28S rDNA	M	*Hoplosternum littorale*	Peru	PV765351	[6]
*Clinostomum chaacci* (L3)	28S rDNA	M	*Rhamdia guatemalensis*	Mexico	MH159730	[30]
*Clinostomum chaacci* (L3)	28S rDNA	M	*Gobiomorus maculatus*	Mexico	MH159731	[30]

^a^ Isolates originally reported under informal names and assigned to *C. chaacci* based on their position within the *C. chaacci* clade in previous studies [1,6] and in the present analysis (Figure 2). M, metacercaria; A, adult.

**Table 2:** Morphometric comparison of *Clinostomum* metacercariae infecting freshwater fishes in Peru.

	*Clinostomum chaacci*	*Clinostomum* sp.	*Clinostomum chaacci*
Host	*Pimelodella cristata*	*Hoplosternum littorale*	*Hoplosternum littorale*
Locality	Loreto Region, Peru.	Ucayali region, Peru	Loreto region, Peru
*n*	5	4	4
Reference	Present study	Chero et al. [6]	Chero et al. [6]
Body length (BL)	3950–4760	4713–5514	4100–6500
Body width (BW)	970–1080	1074–1311	1200–2000
BL/BW	3.9–4.7	3.64–4.39	3.14–3.25
Forebody length	1540–1740	1773–1863	1326–1430
Hindbody length	2410–3010	2706–2980	1902–2435
Collar length	-	231–384	160–200
Collar width	-	603–764	170–250
Oral sucker length (OSL)	222–273	277–296	246–288
Oral sucker width (OSW)	209–258	261–312	278–296
OSW/BW	0.21–0.26	0.22–0.24	-
Pharynx length	120–150	95–114	98–120
Pharynx width	137–158	72–91	76–96
Ventral sucker length (VSL)	624–685	684–758	619–727
Ventral sucker width (VSW)	627–689	693–771	687–743
VSW/BW	0.59–0.67	0.57–0.65	0.37–0.58
VSW/OSW	2.49–3.08	2.47–2.66	2.47–2.51
Distance between suckers	-	640–778	638–679
Anterior testis length (ATL)	216–332	288–324	170–274
Anterior testis width (ATW)	273–419	283–406	379–452
ATW/ATL	1.08–1.26	0.97–1.25	1.64–2.22
Posterior testis length (PTL)	241–297	212–368	195–260
Posterior testis width (PTW)	319–440	344–454	261–515
PTW/PTL	1.30–1.75	1.23–1.71	1.33–1.99
Distance between testes	-	199–271	281–288
Ovary length (OL)	114–170	122–165	130–170
Ovary width (OW)	72–89	83–103	86–110
OW/OL	0.52–0.63	0.61–0.71	0.60–0.70
Cirrus sac length (CSL)	259–294	380–393	390–400
Cirrus sac width (CSW)	166–213	118–171	120–185
CSL/BL	0.05–0.07	0.07–0.08	0.06–0.08

## Data Availability

The sequences obtained in this study are deposited in GenBank under the accession numbers QB134948, QB134949 (*Cox1*) and QB131873 (28S rDNA).

## Supplementary Materials

The following supporting information can be downloaded at https://www.mdpi.com/article/10.3390/ani16213417/s1, Table S1: Pairwise uncorrected p-distances among *Cox1* sequences of *Clinostomum* and outgroup taxa; Table S2: Pairwise uncorrected p-distances among 28S rDNA sequences of *Clinostomum* and outgroup taxa; Table S3: Sequences of Clinostomidae and outgroup taxa used in the phylogenetic analyses of *Cox1* and 28S rDNA, with hosts, localities, GenBank accession numbers and references. References [13,14,15,16,17,18,19,20,21,22,23,24,25,26,27,28,29,30,31,32,33,34,35,36,37,38,39,40,41,42] are cited in the Supplementary Materials.
